# Environmental noise exposure is associated with atherothrombotic risk

**DOI:** 10.1038/s41598-022-06825-0

**Published:** 2022-02-24

**Authors:** Magali Koczorowski, Nadine Bernard, Frédéric Mauny, Frederic Chagué, Sophie Pujol, Maud Maza, Yves Cottin, Marianne Zeller, ENVI-MI Study Group

**Affiliations:** 1 UMR CNRS 6249 Chrono Environnement, Université de Bourgogne Franche Comté, Dijon Cedex, France; 2 uMETh, Inserm CIC 1431, CHU de Besançon, 25030 Besancon Cedex, France; 3 UMR CNRS 6049 ThéMA, Université de Bourgogne Franche Comté, Dijon Cedex, France; 4 Cardiologie, CHU Dijon Bourgogne, Dijon Cedex, France; 5 Equipe Physiolopathologie Et Epidémiologie Cérébro-Cardiovasculaire (PEC2), EA 7460, Université de Bourgogne Franche Comté, Dijon Cedex, France

**Keywords:** Environmental sciences, Cardiology, Risk factors

## Abstract

There is growing evidence that environmental noise exposure could increase the risk of atherothrombotic events, including acute myocardial infarction (MI). We analysed the burden of environmental noise on atherothrombotic risk in MI patients. From the RICO survey, 879 consecutive MI patients included from 2004 to 2008 and living in an urban unit of > 237,000 inhabitants were analysed. Atherothrombotic risk was calculated using the TRS-2P score. TRS-2P categories were split into low (TRS-2P = 0/1) (40.8%), medium–low (TRS-2P = 2) (25.7%), medium–high (TRS-2P = 3) (21.8%) and high risk (TRS-2P ≥ 4) (11.6%). Noise exposure was associated with atherothrombotic risk, with the L_Aeq,24 h_ (OR (95% CI): 1.165 (1.026–1.324)) and L_night_ (OR (95CI): 1.157 (1.031–1.298)), for each 10 dB(A) increase. After adjustment, noise exposure remained a predictor of atherothrombotic risk, with L_Aeq,24 h_ (OR (95% CI): 1.162 (1.011–1.337)) and with L_night_ (OR (95% CI): 1.159 (1.019–1.317)). The relationship with transportation L_night_ was significant for men (OR (95% CI): 1.260 (1.078–1.472)) but not for women (OR (95% CI): 0.959 (0.763–1.205)). We found a significant association between residential traffic noise exposure and atherothrombotic risk in men but not in women. These results could have major consequences for secondary prevention.

## Introduction

Environmental noise has a major impact on health, especially in urban areas where people are continuously exposed to noise from road traffic, railways, and aircrafts. A growing body of evidence has led the World Health Organization (WHO) to declare that exposure to environmental noise can exert non-auditory effects including reduced well-being and quality of life, annoyance, adverse birth outcomes, cognitive impairments, sleep disturbance, metabolic abnormalities, and poorer mental health^1^. In addition, the WHO estimates that the number of DALYs (disability-adjusted life-years) lost as a result of environmental noise in western Europe ranges from 1 to 1.6 million^2^.

Epidemiological studies have also suggested that noise exposure could be associated with an increased risk of cardiovascular (CV) disease including acute myocardial infarction (MI)^3^, and that this association could be even more pronounced in patients with coronary artery disease (CAD). A recent meta-analysis commissioned by the WHO concluded that, from a threshold of 50 dB, road-traffic noise increases the incidence of CAD by 8% per 10 dB(A) (L_den_) (A). This degree of risk, obtained from 7 longitudinal studies, was rated as a high level of evidence^4^. However, only few studies have investigated the specific burden of environmental noise exposure on atherothrombotic risk. Moreover, it remains unclear whether exposure to transportation noise at home could have sex-specific effects since few studies have assessed whether there is a gender-based difference in the effect of noise exposure. In an experimental study, Beheshti et al. found that women experience more annoyance than men when exposed to low-intensity noise^5^. Conversely, men might be more disturbed by traffic noise than women during sleep^6^. An association between hypertension and residential aircraft noise exposure was observed only in men^7,8^. On the contrary, Babisch et al. found no sex-based differences in the association between traffic noise and CV risk^9^.

In the present study, using the database of a regional registry for acute MI and supported by environmental prediction models, we aimed to analyse the burden of residential environmental noise on atherothrombotic risk and to assess the potential differences between men and women.

## Methods

### Study population

The population of this study came from the RICO (obseRvatoire des Infarctus de Côte-d'Or). This ongoing French regional survey prospectively collects data from consecutive patients hospitalized for acute MI in 6 hospitals of one eastern region of France, since 1 January 2001, as previously described^10,11^. Each new episode of MI leads to a new entry in the RICO, so a patient can be registered several times in case of recurrence. Research protocol was performed in accordance with the Declaration of Helsinki. Experimental protocol was approved by ethics committee from CHU Dijon Bourgogne and informed consent was obtained from all subjects.

**Figure 1:**
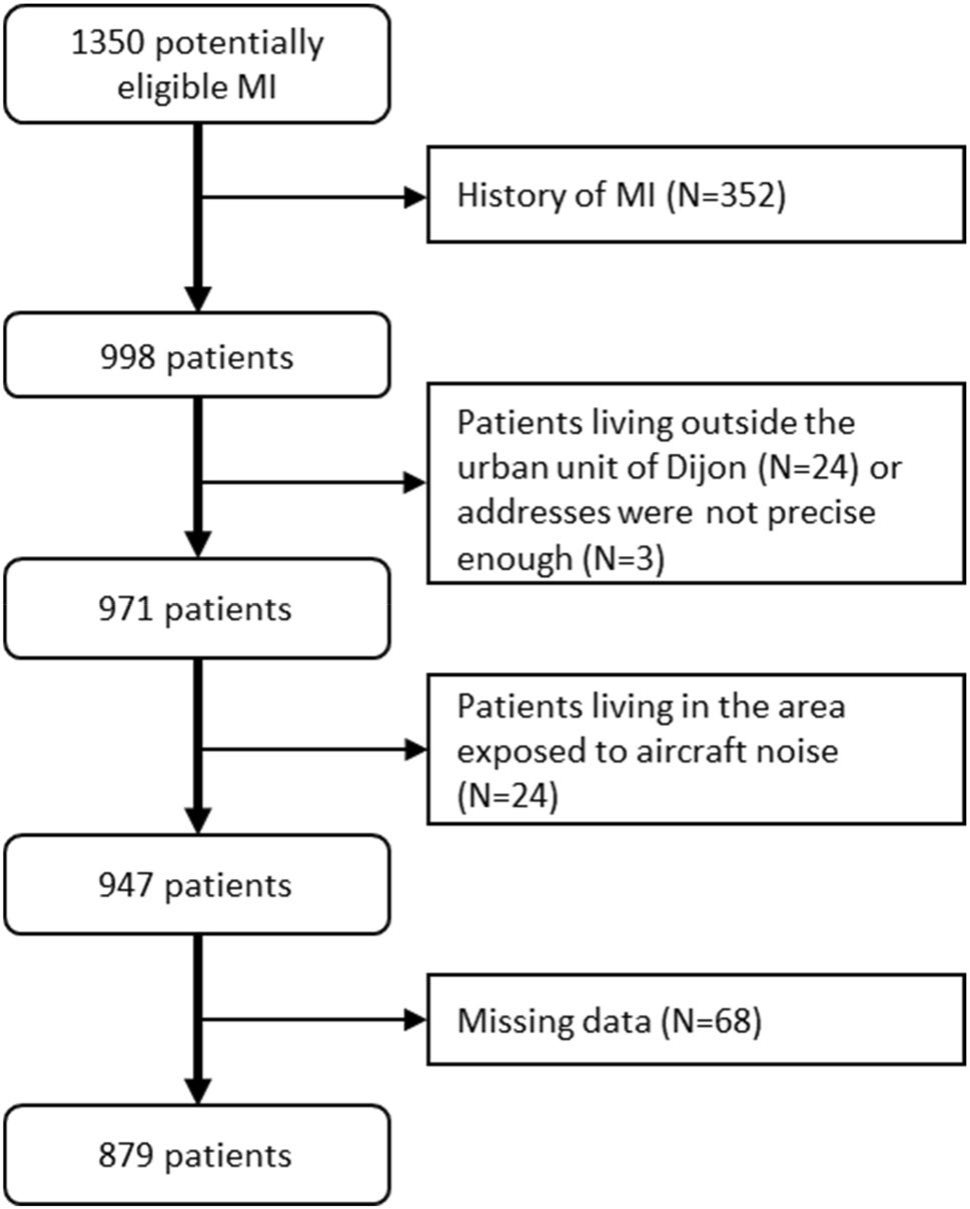
Flow chart of patient selection.

Patients who were admitted for MI for the first time between 1 January 2004 and 31 December 2008 were eligible for the present study (Fig. 1). Inclusion criteria were: living in the urban unit of Dijon (15 towns and 237,000 inhabitants), a known and precise home address at the time of the MI episode. Patients living in an area exposed to the aircraft noise from the military air base of Dijon (2 towns, 200 inhabitants) were excluded because noise data were not available. According to the guidelines of the EU directive, a 5-year period (2004–2008) was selected for the environmental prediction models used for noise exposure.

### Medical data

Clinical, biological and demographic data were collected at hospital admission, including risk factors, comorbidities, chronic medications, heart failure (HF) assessed by Killip class, and hemodynamic parameters. An echocardiography was performed < 12 h after admission to measure left ventricular ejection fraction, as previously reported^10,11^.

The Thrombolysis in Myocardial Infarction Risk Score for Secondary Prevention (TRS-2P) was used to quantify atherothrombotic risk^12^. TRS-2P is calculated by the arithmetic sum of 9 variables associated with atherothrombotic risk: age ≥ 75 years, diabetes mellitus, hypertension, current smoking, peripheral artery disease (PAD), prior stroke, prior coronary artery bypass grafting (CABG), history of HF, and renal failure (estimated glomerular filtration rate < 60 mL/min) (supplementary Fig. 1). TRS-2P was developed in a large population with atherothrombosis to predict recurrent CV events^12^ and has been validated in various populations to characterize long-term risk after recent acute MI^13,14^. The TRS-2P score ranges from 0 to 9 and was split for more risk classification relevance, into 4 classes of atherothrombotic risk namely: low-risk (TRS-2P = 0/1), medium–low-risk (TRS-2P = 2), medium–high-risk (TRS-2P = 3) and high-risk (TRS-2P ≥ 4).

### Environmental noise exposure

According to the European directive 2002/49/EC^15^, environmental noise prediction models were used to assess the outdoor residential exposure of each study patient; these emission-propagation models were elaborated with MITHRA-SIG v3.7 (Geomod/CSTB) software. The models were validated by means of several measurement campaigns^16,17^. Noise levels were calculated for the residential building, in front of each façade and at each floor. They were then linked to the patient through their self-declared home address at the time of their MI. The address of each patient at the time of their MI was carefully gathered and verified to ensure that the assigned building corresponded to the right exposure. For each patient, the average building noise levels in front of the entire façade were calculated. Multiple noises sources were considered: rail traffic, road traffic, and pedestrian traffic. Four indices were computed: road traffic noise and the railway noise (considering only one source, i.e. road or railway respectively), transportation noise (combining road and railway sources), and global noise (combining all the sources). Noise levels were calculated for two time periods, as defined in ISO 1996-2:2017^18^, and expressed as the daily equivalent A-weighted noise level L_Aeq_,_24 h_ and the night equivalent A-weighted noise level L_night_ (22:00–6:00).

### Air pollution exposure: nitrogen dioxide (NO_2_) and particulate matter (PM_10_)

The NO_2_ and PM_10_ outdoor concentrations were calculated using a two-step emission and diffusion modeling^19,20^ First, the daily averaged annual road-traffic emissions were calculated using Circul’Air, software developed and used by the French air quality monitoring network on the basis of the COPERT4 European standard methodology. Second, air pollutant sources from the pollutant emission inventory related to all activity sectors^21^, especially heating, industries or agriculture, were introduced in ADMS-Urban©. This pollution diffusion software relies on state of the art algorithms based on traffic flow, meteorological data, background concentrations and buildings morphology. It has been developed in accordance with the WHO guidelines by the Cambridge Environmental Research Consultants company^22^ and is widely used in Europe for modeling air quality on scales ranging from large urban areas to the street level. The validity of the NO2 and PM10 models were verified using both sampling seasonal measurement campaigns and/or the city's fixed air-quality monitoring network^23^. These two air pollutants concentrations were expressed in µg/m^3^ considering the 3-months period before the occurrence of the MI.

### Socioeconomic level

The neighbourhood socioeconomic level was estimated with a deprivation index based on the French sub-municipal census block groups (called IRIS). As defined by the National Institute of Statistic and Economics Studies, an IRIS (acronym of aggregated units for statistical information) has homogeneous type of habitat and about 2000 inhabitants^20,24^. This index was split into three classes: affluent, mixed and deprived IRIS. The socioeconomic level of each patient was calculated with the R package SesIndexCreator^24^.

### Statistical analysis

Patient characteristics for each sex are summarized as numbers (percentages) for categorical variables and as medians (interquartile range) for continuous variables. Atherothrombotic risk was categorized on 4 classes named: low-risk, medium–low-risk, medium–high-risk, high-risk. The choice of 4 TRS-2P score division was made to balance the number in each risk category, as patients in the highest risk groups were rare (TRS-2P = 6, N = 7; TRS-2P = 7, N = 2), in the absence of consensual discretisation^13,14,25^. Ordinal regressions with adjacent category models were used to assess the relationship between atherothrombotic risk and noise levels. The Brant test was used to test the proportional odds assumption. Variables associated with atherothrombotic risk at a P-value ≤ 0.20 in the analysis were included using a bidirectional step-by-step elimination procedure in the multivariable analysis. Multivariable models were then adjusted for sex, socio-economic level and heart rate; in the analysis stratified on sex, the multivariable model was only adjusted for socio-economic level and heart rate. Sensitivity analyses were performed with the TRS-2P score in 8 levels, with cumulative logit models and by stratifying the age into two groups (threshold 60 y for men, 65 y for women). Odds ratios associated to noise exposure were expressed for an increase of 10 dB(A). Statistical analysis was performed using R version 4.0.2 statistical software^26^. Sensitivity analyses were further performed using polytomous logistic regression and cumulative ordinal logistic regression.

## Results

Among the 1,350 eligible patients, 879 patients were included in the study (Fig. 1). Baseline characteristics of the population are shown in Table 1. Most patients were men (n = 567, 64.5%). The highest risk group of the TRS-2P score did not include any women. Women were on average 10 years older than men (74 vs 64 y). Hypertension and renal failure were more frequent among women (p < 0.001). There were more male current smokers (p < 0.001), and men had higher diastolic blood pressure on admission than women (p < 0.001).

**Table 1:** Population characteristics (N(%) or median(IQR)).

N (%) or Median (IQR)	Men (N = 567)	Women (N = 312)	Total (N = 879)
**Patient characteristics**			
Age (years)	64 (54–76)	78 (67–85)	70 (57–80)
**Age (classes)**^**a**^			
< 75	404 (71.3%)	121 (38.8%)	525 (59.7%)
≥ 75	163 (28.7%)	191 (61.2%)	354 (40.3%)
BMI (kg/m^2^)	26 (24–29)	25 (22–29)	26 (24–29)
Obesity (BMI ≥ 30)	82 (14.5%)	48 (15.8%)	130 (15.0%)
Hypertension^a^	264 (46.6%)	216 (69.2%)	480 (54.6%)
Diabetes mellitus^a^	117 (20.6%)	74 (23.7%)	191 (21.7%)
Hypercholesterolemia	245 (43.5%)	145 (46.9%)	390 (44.7%)
Current smoking^a^	195 (34.4%)	43 (13.8%)	238 (27.1%)
**Socio-economic level**^**b**^			
Affluent	149 (26.3%)	70 (22.4%)	219 (24.9%)
Mixed	348 (61.4%)	211 (67.6%)	559 (63.6%)
Deprived	70 (12.3%)	31 (9.9%)	101 (11.5%)
**Comorbidities**			
Previous CAD	36 (6.3%)	29 (9.3%)	65 (7.4%)
Family history of CAD	130 (23.4%)	94 (30.9%)	224 (26.1%)
Prior stroke^a^	29 (5.1%)	29 (9.3%)	58 (6.6%)
PAD^a^	46 (8.1%)	22 (7.1%)	68 (7.7%)
Prior CABG^a^	5 (0.9%)	5 (1.6%)	10 (1.1%)
Congestive heart failure^a^	23 (4.1%)	11 (3.5%)	34 (3.9%)
COPD	27 (4.8%)	15 (4.8%)	42 (4.8%)
Renal failure^a^	157 (27.7%)	144 (46.2%)	301 (34.2%)
**Clinical data**			
Heart rate (beat/min)^a^	78 (64–90)	80 (68–96)	79 (66–92)
Systolic blood pressure (mmHg)	140 (123–160)	141 (124–165)	140 (123–160)
Diastolic blood pressure (mmHg)	83 (71–95)	80 (70–90)	80 (70–93)
LVEF (%)	55 (46–65)	54 (45–63)	55 (45–65)
LVEF ≤ 40%	89 (17.7%)	59 (21.1%)	148 (18.9%)
STEMI	311 (54.9%)	158 (50.6%)	469 (53.4%)
Anterior wall location	186 (32.8%)	127 (40.7%)	313 (35.6%)
**Killip class on admission**			
1	474 (83.6%)	228 (74.0%)	702 (80.2%)
2	63 (11.1%)	55 (17.9%)	118 (13.5%)
3	26 (4.6%)	24 (7.8%)	50 (5.7%)
4	4 (0.7%)	1 (0.3%)	5 (0.6%)
**Chronic medications**			
Aspirin	69 (13.0%)	51 (17.6%)	120 (14.7%)
Statin	101 (19.1%)	67 (23.1%)	168 (20.5%)
**TRS-2P score**			
**Low risk**			
0	82 (14.5%)	25 (8.0%)	107 (12.2%)
1	200 (35.3%)	52 (16.7%)	252 (28.7%)
**Low medium risk**			
2	144 (25.4%)	82 (26.3%)	226 (25.7%)
**High medium risk**			
3	88 (15.5%)	104 (33.3%)	192 (21.8%)
**High risk**			
4	32 (5.6%)	33 (10.6%)	65 (7.4%)
5	15 (2.6%)	13 (4.2%)	28 (3.2%)
6	4 (0.7%)	3 (1.0%)	7 (0.8%)
7	2 (0.4%)	0 (0.0%)	2 (0.2%)

There were no significant differences in baseline characteristics between men and women.

*BMI* body mass index, *CABG* coronary artery bypass graft, *CAD* coronary artery disease, *COPD* chronic obstructive pulmonary disease, *IQR* interquartile range, *LVEF* left ventricular ejection fraction, *MI* myocardial infarction, *N* number, *PAD* peripheral artery disease, *STEMI* ST-segment elevation myocardial infarction.

^a^Items used to calculate the TRS-2P score (Thrombolysis in Myocardial Infarction Risk Score for Secondary Prevention).

^b^Deprivation index (19).

**Figure 2:**
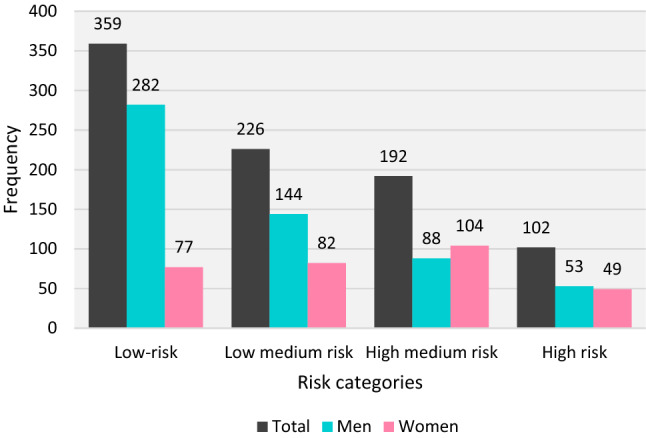
Distribution of risk categories of TRS-2P score: low-risk, TRS-2P = 0/1; medium–low-risk, TRS-2P = 2; medium–high-risk, TRS-2P = 3; and high-risk, TRS-2P ≥ 4.

**Figure 3:**
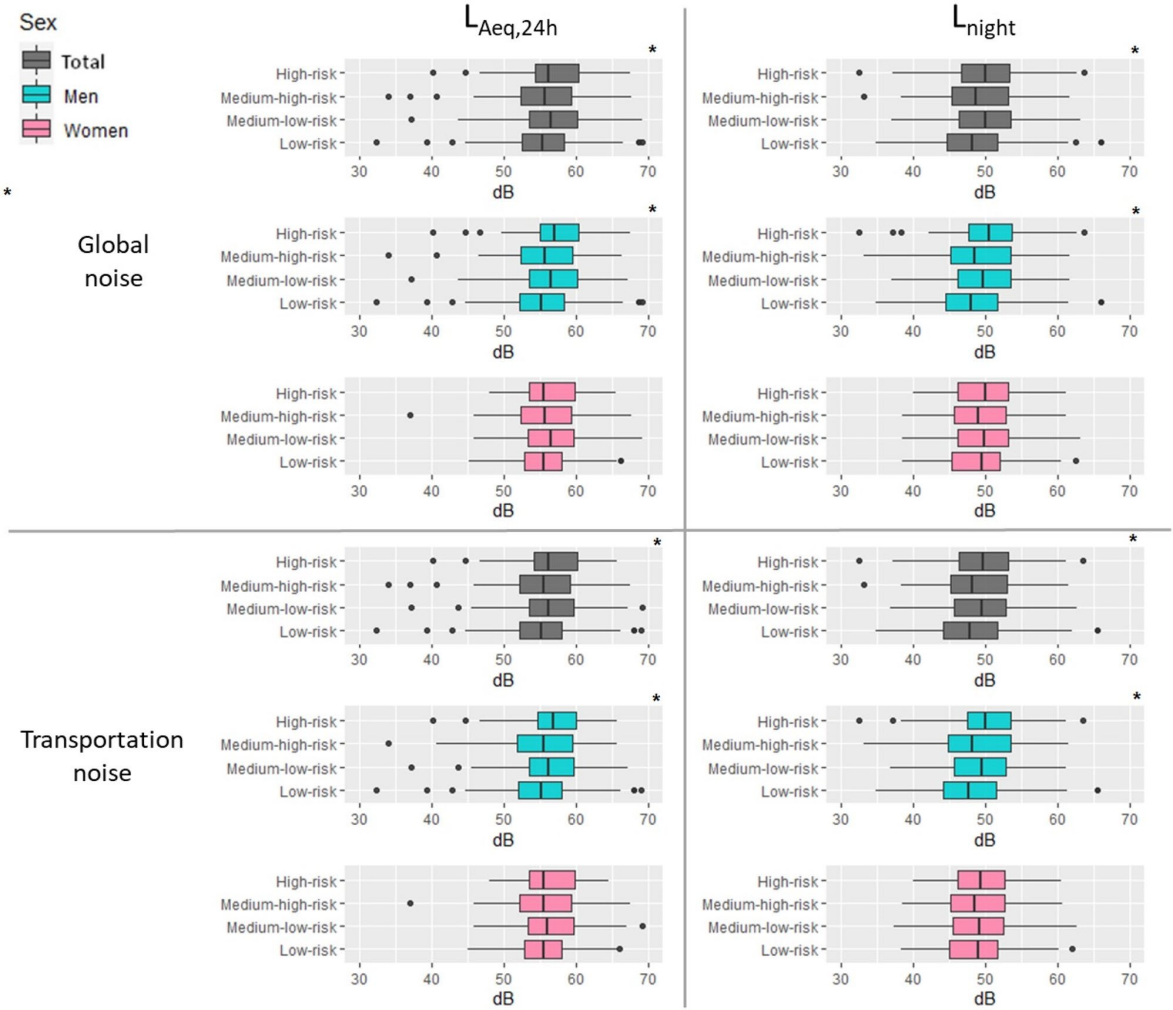
Levels of global and transportation noise exposure, for 24 h and at night, in men and women. *p-value < 0.05, model adjusted on socio-economic level and cardiac frequency (Model 1).

**Figure 4:**
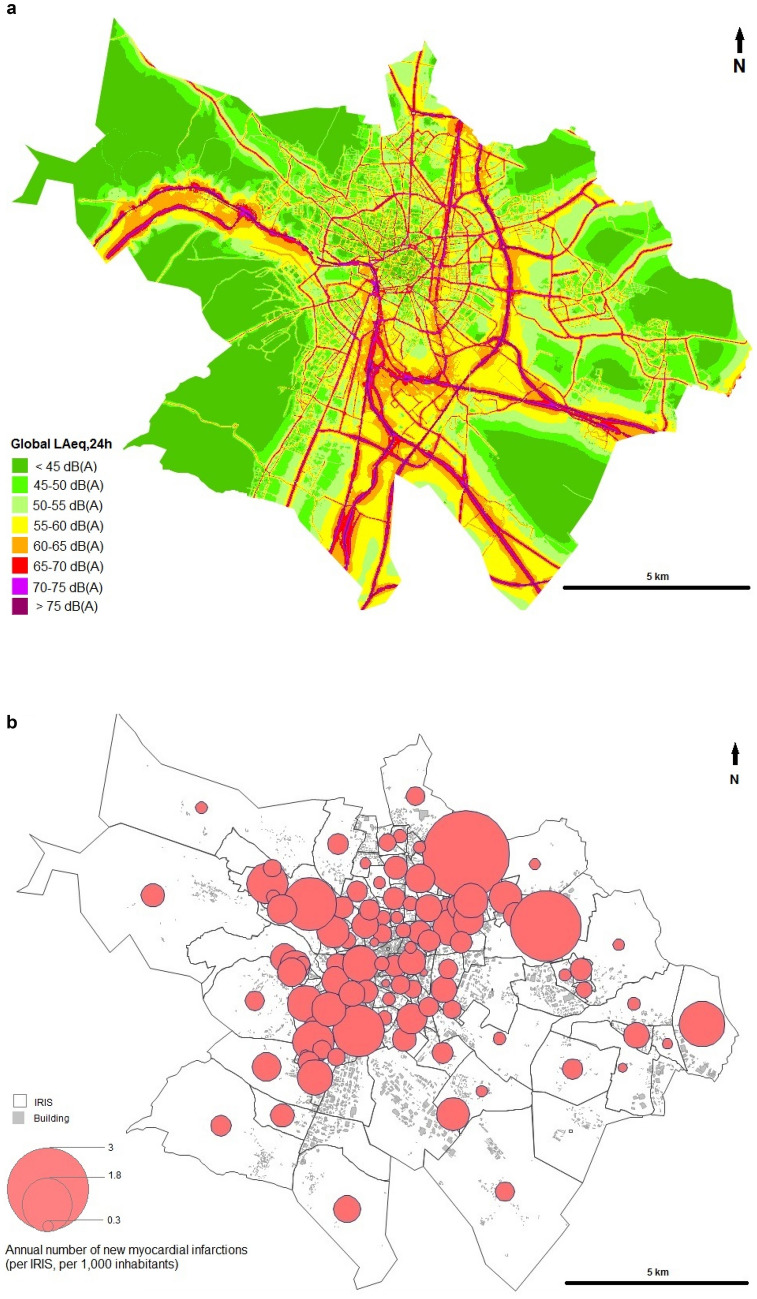
Environmental noise map (global L_Aeq,24 h_) and spatial distribution of the observed cases of myocardial infarction. (**a**) Environmental Noise map (global L_Aeq,24 h_). (**b**) Annual number of new myocardial infarctions according to the IRIS population size (per 1000 inhabitants).

Nearly half of men (N = 282, 49.7%) were in the low-risk category, while the majority of women (N = 104, 33.3%) were in medium–high-risk category (Table 1, Fig. 2). The levels of environmental exposure are presented in supplementary Table 1. Not surprisingly, the L_Aeq,24 h_ were higher than the L_night_ (Fig. 3 and supplementary Table 1). An environmental noise map (global L_Aeq,24 h_) and the spatial distribution of the observed cases of myocardial infarction are presented in Fig. 4. The score criteria and the distribution of each of the items used in the TRS-2P according to the four atherothrombotic risk classes are shown in supplementary Figs. 2 and 3. Among the components of the risk score, hypertension was the main driver of atherothrombotic risk, except in the low-risk group, where smoking was the most frequent (supplementary Figs. 2–3).

Noise exposure was significantly associated with atherothrombotic risk for all the noise sources and the L_Aeq,24 h_ and L_night_ in univariable analysis. The odds ratio associated with a 10 dB(A) increase in road-related noise and railway-related noise were 1.161 (95% CI 1.021–1.320, P = 0.024) and 1.028 (95% CI 1.000–1.057, P = 0.049), respectively. In multivariable analysis (model 1), the association was only weakly modified after adjustment for global and traffic noise over 24 h and at night, for an increase of 10 dB(A), and most associations remained significant or nearly significant (Table 2). After adjustment on air pollutant exposure (model 1 + PM_10_ or model 1 + NO_2_), the observed OR increased and the associations remained significant.

**Table 2:** Associations between noise exposure and risk categories of TRS-2P score.

	Univariate		Adjusted model 1^a^		Model 1 + NO_2_		Model 1 + PM_10_	
	OR (95% CI)	P-value	OR (95% CI)	P-value	OR (95% CI)	P-value	OR (95% CI)	P-value
**L**_**Aeq,24 h**_								
Global	**1.165 (1.026–1.324)**	0.019	**1.162 (1.011–1.337)**	0.036	**1.229 (1.014–1.491)**	0.037	**1.216 (1.010–1.463)**	0.039
Transportation	**1.164 (1.024–1.324)**	0.021	**1.160 (1.009–1.334)**	0.038	**1.223 (1.008–1.485)**	0.042	**1.210 (1.005–1.458)**	0.044
Railway only	**1.028 (1.000–1.057)**	0.049	1.026 (0.994–1.058)	0.109	1.040 (0.999–1.082)	0.056	1.040 (1.000–1.081)	0.053
Roads only	**1.161 (1.021–1.320)**	0.024	1.140 (0.993–1.310)	0.065	1.128 (0.932–1.365)	0.217	1.121 (0.933–1.346)	0.226
**L**_**night**_								
Global	**1.157 (1.031–1.298)**	0.013	**1.159 (1.019–1.317)**	0.025	**1.239 (1.045–1.469)**	0.014	**1.228 (1.040–1.448)**	0.016
Transportation	**1.157 (1.031–1.298)**	0.014	**1.161 (1.022–1.320)**	0.022	**1.248 (1.052–1.479)**	0.011	**1.236 (1.048–1.459)**	0.012
Railway only	**1.030 (1.001–1.060)**	0.043	1.028 (0.995–1.062)	0.095	**1.043 (1.001–1.087)**	0.046	**1.043 (1.001–1.087)**	0.044
Roads only	**1.166 (1.026–1.325)**	0.019	1.147 (0.998–1.319)	0.054	1.139 (0.940–1.379)	0.185	1.131 (0.940–1.360)	0.194

Odds ratios associated to noise exposure expressed for an increase of 10 dB(A).

*OR* odds ratio, *CI* confidence interval.

Significant values are in bold.

^a^Model 1: Model adjusted on sex, socio-economic level and heart rate.

The analyses of the interactions between noise exposure and sex are presented in supplementary Table 2. In stratified analysis, the associations remained significant in men (Table 3), but were no longer found in women (Table 4). Global noise, and the transportation and railway noise levels were significantly associated with an increase in atherothrombotic risk among men for both periods. For men, the odds associated with an increase of 10 dBA in transportation L_night_ was 1.260 (95% CI 1.078–1.472, P = 0.004) (model 1). After adjustment on air pollutant exposure (model 1 + PM_10_ or model 1 + NO_2_), the observed OR weakly decreased and the associations remained significant in model 1 + PM_10_. For women, for an increase of 10 dBA in transportation L_night,_, the OR was 0.959 (95% CI 0.763–1.205, P = 0.721) Adjustment on air pollutants lead to the same results.

**Table 3:** Associations between noise exposure and risk categories of TRS-2P score, stratified by sex: men’s table.

Men	Univariate model		Adjusted model 1^a^		Model 1 + NO_2_		Model 1 + PM_10_	
	OR (95% CI)	P-value	OR (95% CI)	P-value	OR (95% CI)	P-value	OR (95% CI)	P-value
**L**_**Aeq24h**_								
Global	**1.246 (1.057–1.470)**	0.009	**1.244 (1.047–1.479)**	0.014	1.216 (0.996–1.484)	0.056	**1.228 (1.019–1.480)**	0.032
Transportation	**1.243 (1.052–1.468)**	0.011	**1.239 (1.041–1.473)**	0.016	1.208 (0.989–1.475)	0.066	**1.221 (1.013–1.472)**	0.037
Railway only	**1.045 (1.008–1.083)**	0.017	**1.048 (1.009–1.089)**	0.016	1.040 (0.999–1.082)	0.056	**1.042 (1.003–1.084)**	0.037
Roads only	1.169 (0.990–1.379)	0.067	1.148 (0.966–1.364)	0.120	1.094 (0.900–1.331)	0.369	1.120 (0.932–1.346)	0.228
**L**_**night**_								
Global	**1.248 (1.077–1.446)**	0.003	**1.260 (1.078–1.472)**	0.004	**1.240 (1.041–1.478)**	0.017	**1.248 (1.057–1.474)**	0.009
Transportation	**1.257 (1.084–1.457)**	0.003	**1.265 (1.083–1.478)**	0.003	**1.247 (1.047–1.485)**	0.014	**1.255 (1.062–1.482)**	0.008
Railway only	**1.048 (1.010–1.088)**	0.014	**1.052 (1.011–1.094)**	0.013	**1.043 (1.001–1.087)**	0.046	**1.046 (1.004–1.089)**	0.031
Roads only	1.179 (0.999–1.390)	0.053	1.159 (0.973–1.381)	0.099	1.108 (0.910–1.350)	0.310	1.133 (0.941–1.364)	0.190

Odds ratios associated to noise exposure expressed for an increase of 10 dB(A).

*OR* odds ratio, *CI* confidence interval.

Significant values are in bold.

^a^Model 1: Model adjusted on socio-economic level and cardiac frequency.

**Table 4:** Associations between noise exposure and risk categories of TRS-2P score, stratified by sex, women’s table.

Women	Univariate model		Adjusted model 1^a^		Model 1 + N0_2_		Model 1 + PM_10_	
	OR (95% CI)	P-value	OR (95% CI)	P-value	OR (95% CI)	P-value	OR (95% CI)	P-value
**L**_**Aeq24h**_								
Global	1.034 (0.825–1.295)	0.773	1.013 (0.796–1.290)	0.914	1.004 (0.767–1.315)	0.976	0.952 (0.733–1.236)	0.711
Transportation	1.039 (0.829–1.302)	0.740	1.020 (0.802–1.297)	0.873	1.014 (0.776–1.325)	0.920	0.961 (0.741–1.247)	0.766
Railway only	0.995 (0.948–1.043)	0.822	0.979 (0.927–1.035)	0.459	0.969 (0.916–1.026)	0.285	0.966 (0.913–1.022)	0.236
Roads only	1.132 (0.904–1.418)	0.282	1.133 (0.895–1.434)	0.301	1.181 (0.906–1.541)	0.222	1.106 (0.857–1.427)	0.441
**L**_**night**_								
Global	0.997 (0.811–1.226)	0.977	0.959 (0.763–1.205)	0.721	0.923 (0.719–1.186)	0.535	0.882 (0.688–1.13)	0.323
Transportation	0.997 (0.811–1.226)	0.976	0.964 (0.770–1.208)	0.752	0.931 (0.729–1.189)	0.569	0.891 (0.698–1.137)	0.355
Railway only	0.994 (0.946–1.045)	0.820	0.979 (0.924–1.037)	0.465	0.968 (0.913–1.027)	0.288	0.965 (0.909–1.024)	0.237
Roads only	1.136 (0.909–1.420)	0.264	1.136 (0.899–1.436)	0.287	1.179 (0.907–1.531)	0.221	1.108 (0.860–1.426)	0.429

Odds ratios associated to noise exposure expressed for an increase of 10 dB(A).

*OR* odds ratio, *CI* confidence interval.

^a^Model 1: Model adjusted on socio-economic level and cardiac frequency.

Sensitivity analyses were further performed using (i) polytomous logistic regression (ii) cumulative ordinal logistic regression and then (iii) groups stratified by age. The associations remained significant among men and non-significant among women.

## Discussion

Our findings, obtained using data from a population of high-risk patients, show that exposure to environmental noise, in particular from transportation traffic, was associated with atherothrombotic risk. This relation was sex specific seeing as it was only observed in men.

The baseline characteristics of our study population, including sex ratio and rate of major risk factors, were similar to the general French MI population^25^, although our population was slightly younger (70.0 y vs 66.0 y, respectively). The distribution of low-/intermediate-/high-risk levels in our study was also similar (41%/26%/33% vs 42%/24%/34%)^13^. These data suggest the absence of a major selection bias in our study population and further reinforce the relevance of our findings. One strength of this study was the precise attribution of residential noise exposure, which was directly associated with geocoded patient addresses using a reproducible process completely unrelated to medical status. The acoustic levels in this city could be considered as moderate, The urban unit of Dijon is a ‘medium sized’ European city (i.e. city of 100,000 to 500,000 inhabitants)^27^. Over 44% of the European population is living in this category of cities^28^. The deprivation index was a collective measurement of neighbourhood socioeconomic status. It was defined using an accurate and reproducible procedure^24^, and it was thus possible to adjust for a potential influence of socio-economic factors in the studied relationships. Two pollutants related to road traffic were studied. NO_2_ is a gaseous pollutant known to be the main indicator of road traffic^29^. Particulate matters (PM_10_) are also generated by road traffic and residential heating and were chosen because of their significant impact on human^30,31^.

To the best of our knowledge, our study is the first to focus on the relationship between noise and atherothrombotic risk in high-risk patients. TRS-2P, a risk stratification tool, was developed to identify patients with a higher relative risk who are likely to benefit from the personalised administration of preventive measures. In real world patients, it was recently found to predict long-term outcomes, in particular for recurrent events^14^. Patients in elevated risk categories (TRS-2P score ≥ 5) had a dramatically increased risk of major cardiac events, in particular for recurrent MI (HR (95% CI) 10.03 (8.52–11.81)), when compared with lowest score (TRS-2P score = 0)^32^. Our data thus also suggests that chronic residential exposure could be associated with an increased risk of future recurrent ischemic events. However, further population-based studies are needed to support this hypothesis. Our decision to divide the TRS-2P score into 4 categories was made to balance the number in each risk category, since patients in the highest risk groups were rare (TRS-2P = 6, N = 7; TRS-2P = 7, N = 2). Furthermore, there is no consensus in the literature for any score threshold. Puymirat et al*.* chose to cut the score into 3 categories (0–1, 2, ≥ 3)^13^ whereas Bergmark et al*.* divided it into 4 groups (≤ 2, 3, 4, and ≥ 5)^33^ and Zafrir et al*.* into 6 (0, 1, 2, 3, 4, and ≥ 5)^14^.

### Noise and CV diseases

Traffic noise is found to be associated with an increased risk of CV diseases, including MI and stroke^34,35^, and one meta-analysis has found that there is a linear relationship between exposure to transport noise and the incidence of ischemic heart disease. Nocturnal noise exposure has been shown to increase heart rate and blood pressure through activation of the sympathetic nervous system, reducing heart rate variability^36,37^. Moreover, associations between road traffic noise and CV diseases were found to be stronger among subjects sleeping with open windows^38^. Of course, we should assume that hypertension is the main cause of atherothrombotic risk, and a contributing factor for almost all patients in the high-risk group. However, the HYENA study also found an association between night-time aircraft noise and excess risk of hypertension (OR = 1.14, 95% CI 1.01–1.29)^8^. A meta-analysis also confirmed that there is a positive association between road traffic noise and hypertension^39^. Noise-induced annoyance could be one of the main contributors to the relationship between noise exposure and hypertension^40^.

### Sex-specific sensitivity to transportation noise

Our results suggest that men are particularly sensitive to environmental noise, and especially to transportation noise. Only few studies have analyzed the influence of sex on the health effects of noise^41^. In experimental contexts, women felt more annoyance than men when exposed to all frequencies of low-intensity noise^5^. However, Röösli et al*.* suggested that men could be more sensitive to traffic-noise-induced sleep disturbance^6^. Sleep duration in highly exposed men (> 55 dB) was reduced by 1.5 h compared with men who had low exposure (< 30 dB), while sleep duration in women was not affected^6^. Convergent data from the HYENA (Hypertension and Exposure to Noise near Airports) study on a large population of 4721 Swedish subjects followed for 8–10 years also suggest that the risk of hypertension associated with residential aircraft noise exposure was significantly increased in men but not in women (RR (95% CI) 1.21 (1.05–1.39), and 0.97 (0.83–1.13), respectively)^7^ . Moreover, in 4,861 persons 45–70 years of age, who had lived at least 5 years near 6 major European airports found significant relationship between daily road traffic noise exposure and risk of hypertension, which was stronger for men (OR of 1.54 (95% CI 0.99–2.40) in the highest exposure category (> 65 dB; p(trend) = 0.008)^8^. The recent DEBATS study also identified this association in men (OR = 1.34, 95% CI 1.00–1.97)^42^. Nonetheless, sex-specific sensitivity remains controversial^9^.

### Underlying pathophysiological mechanisms

Experimental studies addressing the underlying mechanisms for noise-induced CV risk in animal or human are rare. Most works support the hypothesis of a causal relationship, through NADPH oxidase-induced vascular oxidative stress, exacerbation of hypertension, and promotion of pro-thrombotic and proinflammatory plasma phenotype^43–45^ and there is growing evidence from translational studies on how noise may trigger CV effects. In healthy adults, night-time exposure to aircraft noise was associated in a dose-dependent manner with impaired endothelial function^46^. In addition, night-time traffic noise has been shown to cause elevated levels of stress hormones such as cortisol and catecholamine, and sympathetic tone activation^3^. These factors could promote the development of vascular dysfunction (endothelial dysfunction) and high blood pressure, which, in turn, could elevate CV risk. Animal studies have also suggested that noise exposure during sleep causes a more pronounced inflammatory and oxidative stress response than wake-phase exposure^47^. However, some human studies are inconclusive^48^. Whether the extra-auditory effects of noise result directly from exposure or from sleep disturbance and related mental health problems, such as anxiety and depression, which are known CV risk factors, remains to be investigated.

### Study limitations

Our study has some limitations. One is the use of TRS-2P, which includes the most important CV risk factors, as an index of atherothrombotic risk because it limited the relevance of adjustment on other variables. However, although the sample size was small, the odd ratios were only slightly modified by adjustments, highlighting the constancy of the associations between environmental noise and CV risk. We however acknowledge a weaker association between noise and the atherothrombotic risk when considering simultaneously the NO_2_ exposure. The lack of biological sampling to assess CV risk factors such as proinflammatory or prothrombotic markers, limited the conclusion on the link between the CV risk and noise. Furthermore, medical data collected at the time of admission does not represent baseline condition, whereas the noise levels assigned to each patient correspond to chronic exposure. Also, noise exposure levels were assigned to self-declared home address at the time of MI, although some individuals may have moved in the previous months. However, a recent study showed that the rate of moving in the French population was low (i.e. 11%)^49^, and older subjects, mainly retirees, were even less likely to move (9%). Therefore, considering the median age of our study population (70 y), it is likely that only few subjects moved and may thus have been assigned with erroneous levels of noise. People spend 80% of their time indoors^50^, and we did not take into account indoor noise. As this is a retrospective study, noise levels were not measured but modeled, so there is potential uncertainty regarding the validity environmental data. Differences in residential characteristics, such building insulation, were not recorded. Although subjects could spend part of their time out of their home, the noise exposure used was assigned at the home address. The potential errors of the L_night_ estimate should therefore be smaller than for the L_Aeq,24 h_, but our results were similar for L_Aeq,24 h_ and L_night_, which is in favor of a minor error of estimation. No questionnaire on sleeping habits, open or closed windows, sleeping room localization was available. The subject addresses contained only the street name and number. This could have limited the precision of the noise exposure assessment. Only global L_Aeq,24 h_ (dB(A)) map is presented, as noise map for nocturnal exposure could not be generated. However, LAeq,24 h usually closely correlates with Lnight.

## Conclusion

Our results suggest, for the first time, that there is an association between transportation noise exposure and atherothrombotic risk, and they support the hypothesis that men are particularly sensitive to CV effects of chronic noise exposure. Further population-based prospective cohort studies are thus needed to better understand the interactions between sex and the CV effects of environmental noise, and to adapt, if necessary, public health messages.

## Supplementary Information

Supplementary Information.
