# Supplementary material for: Environmental noise exposure is associated with atherothrombotic risk
Source: Sci Rep. 2022 Feb 24;12:3151. doi: 10.1038/s41598-022-06825-0 (PMC8873564; doi:10.1038/s41598-022-06825-0)
Supplement: Supplementary file 1 — Supplementary Information. [file 41598_2022_6825_MOESM1_ESM.docx]

# Supplementary files

**Supplementary Table 1**: Noise and air pollution exposure levels according to sex

|  | **Men** | | | | **Women** | | | | **Total** | | | |
| --- | --- | --- | --- | --- | --- | --- | --- | --- | --- | --- | --- | --- |
|  | **Min** | **Max** | **Mean** | **SD** | **Min** | **Max** | **Mean** | **SD** | **Min** | **Max** | **Mean** | **SD** |
| **Noise exposure** |  | | | |  | | | |  | | | |
| **L_Aeq24h_** |  |  |  |  |  |  |  |  |  |  |  |  |
| Global | 37.0 | 69.2 | 56.0 | 4.8 | 32.4 | 69.2 | 55.9 | 5.1 | 32.4 | 69.2 | 55.9 | 5.0 |
| Roads only | 37.0 | 69.2 | 55.0 | 4.8 | 32.4 | 69.0 | 54.8 | 5.0 | 32.4 | 69.2 | 54.8 | 5.0 |
| Railway only | 0.0 | 65.0 | 28.7 | 22.8 | 0.0 | 67.8 | 27.2 | 23.3 | 0.0 | 67.8 | 27.7 | 23.1 |
| Transportation | 37.0 | 69.2 | 55.9 | 4.8 | 32.4 | 69.0 | 55.7 | 5.1 | 32.4 | 69.2 | 55.8 | 5.0 |
| **L_night_** |  |  |  |  |  |  |  |  |  |  |  |  |
| Global | 29.3 | 63.1 | 49.2 | 5.2 | 24.6 | 65.9 | 48.9 | 5.7 | 24.6 | 65.9 | 49.0 | 5.6 |
| Roads only | 29.3 | 61.4 | 46.9 | 4.9 | 24.6 | 61.1 | 46.7 | 5.1 | 24.6 | 61.4 | 46.8 | 5.0 |
| Railway only | 0.0 | 62.4 | 27.3 | 21.8 | 0.0 | 65.5 | 25.8 | 22.4 | 0.0 | 65.5 | 26.3 | 22.2 |
| Transportation | 29.3 | 62.7 | 48.8 | 5.2 | 24.6 | 65.5 | 48.7 | 5.7 | 24.6 | 65.5 | 48.7 | 5.5 |
| **Air pollution exposure** |  | | | |  | | | |  | | | |
| NO_2_ | 14.5 | 48.5 | 27.1 | 7.3 | 12.7 | 48.3 | 26.5 | 7.1 | 12.7 | 48.5 | 26.7 | 7.2 |
| PM_10_ | 11.6 | 26.5 | 17.7 | 2.8 | 11.3 | 27.6 | 17.5 | 2.7 | 11.3 | 27.6 | 17.6 | 2.8 |

**Supplementary Table 2**: Factor interaction between sex and noise

|  |  | **P-value** | **OR (95% CI)** |
| --- | --- | --- | --- |
| **L_Aeq,24h_** |  |  |  |
| Global |  | 0.185 | 0.829 (0.629-1.092) |
| Transportation |  | 0.207 | 0.836 (0.634-1.103) |
| Railway only |  | **0.082** | 0.949(0.895-1.006) |
| Roads only |  | 0.884 | 0.977(0.744-1.291) |
| **L_night_** |  |  |  |
| Global |  | **0.071** | 0.793(0.617-1.019) |
| Transportation |  | **0.065** | 0.789(0.614-1.014) |
| Railway only |  | **0.075** | 0.945(0.889-1.005) |
| Roads only |  | 0.855 | 0.975(0.741-1.182) |

Abbreviations: OR, odds ratios; CI, confidence interval

**Supplementary Figure 1:** Athero**t**hrombosis risk scoring

| **TRS-2P criteria** | **Points** |
| --- | --- |
| Congestive heart failure | 1 |
| Hypertension | 1 |
| Age≥75 y | 1 |
| Diabetes | 1 |
| Prior stroke | 1 |
| Prior CABG | 1 |
| Peripheral artery disease | 1 |
| Current smoking | 1 |
| eGFR<60 | 1 |
| **Maximum score** | **9** |

**Supplementary Figure 2:** Distribution of items of the TRS-2P across the 4 atherothrombotic risk classes, in men and women


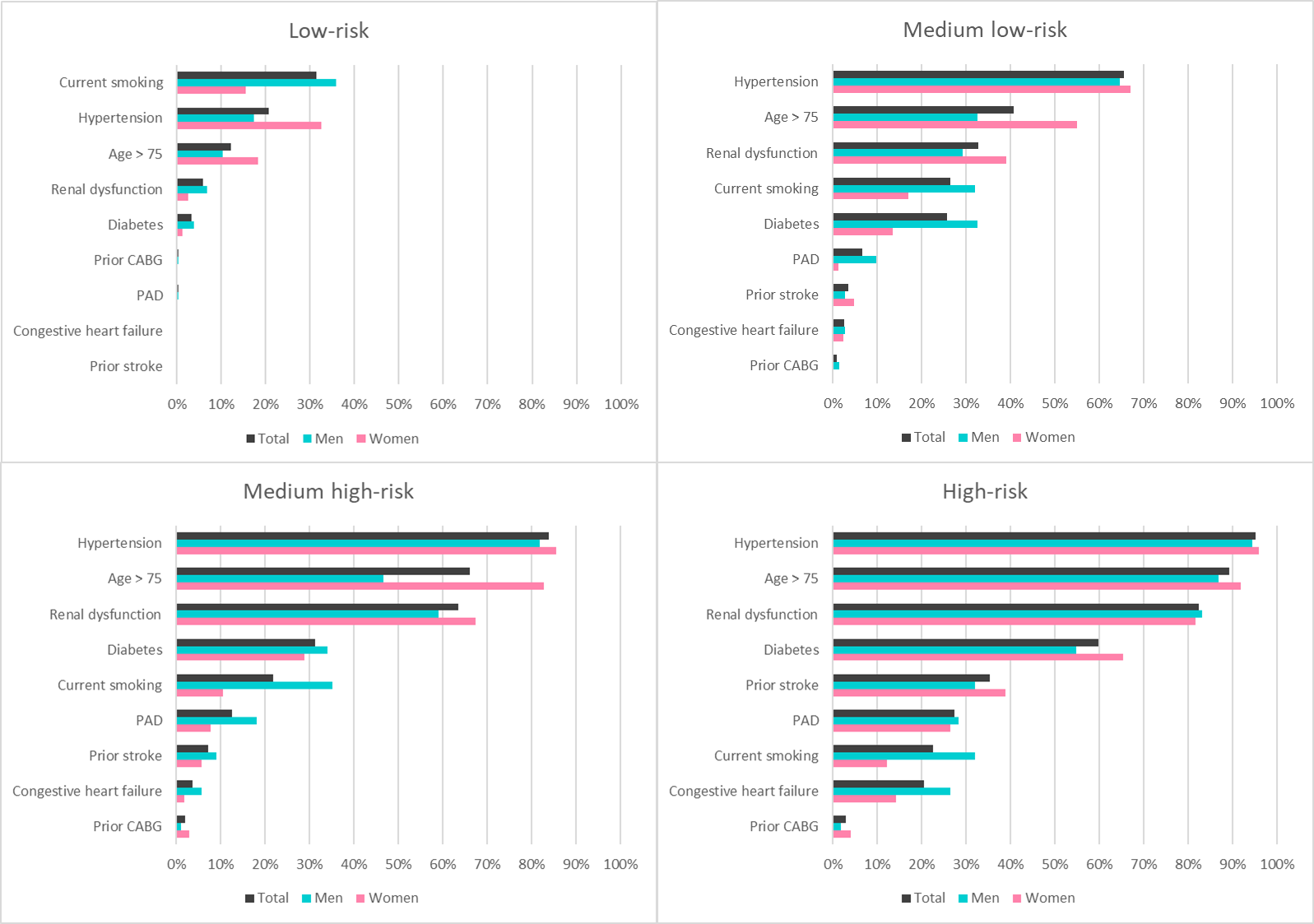
Abbreviations: CABG, coronary artery bypass graft; PAD, peripheral artery disease

**Supplementary Figure 3:** Distribution of items of the TRS-2P in the 4 atherothrombotic risk classes

Abbreviations: CABG, coronary artery bypass graft; PAD, peripheral artery disease
